# Impact of a Multimodal Infection Control Intervention on Central Line-Associated Bloodstream Infections in the ICU

**DOI:** 10.3390/antibiotics15050504

**Published:** 2026-05-18

**Authors:** Hyemin Chung, Insoon Choi, Kye Won Choe, Moonsuk Bae, Joung Ha Park, Oh Joo Kweon, Min-Chul Kim

**Affiliations:** 1 Division of Infectious Disease, Department of Internal Medicine, Chung-Ang University Gwangmyeong Hospital, Chung-Ang University College of Medicine, Gwangmyeong-si 14353, Republic of Korea; hyeminchung@cauhs.or.kr (H.C.); carukeion@gmail.com (M.B.); pjha89@cauhs.or.kr (J.H.P.); 2 Infection Control Office, Chung-Ang University Gwangmyeong Hospital, Gwangmyeong-si 14353, Republic of Korea; ssoony123@cauhs.or.kr (I.C.); choekw@cauhs.or.kr (K.W.C.); poipoi9@cau.ac.kr (O.J.K.); 3 Department of Laboratory Medicine, Chung-Ang University Gwangmyeong Hospital, Chung-Ang University College of Medicine, Gwangmyeong-si 14353, Republic of Korea

**Keywords:** CLABSI, infection control intervention, incidence, device utilization ratio

## Abstract

**Background/Objectives**: Central line-associated bloodstream infection (CLABSI) remains a major healthcare-associated infection in intensive care units (ICUs). This study evaluated changes in CLABSI incidence following the implementation of a multimodal infection control intervention in the ICU. **Methods**: We conducted a quasi-experimental study in the adult ICUs of a referral hospital from January 2023 to December 2025. The interventions included staff education, performance feedback, infection control-led rounds, optimization of catheter practices, and reinforcement of environmental hygiene. The primary outcome was CLABSI incidence per 1000 central line-days. An interrupted time-series analysis using segmented Poisson regression with robust standard errors was used to assess temporal trends. **Results**: A total of 17 CLABSI cases occurred during the pre-intervention period, and 25 during the post-intervention period. There was no significant difference in CLABSI incidence between the two periods (incidence rate ratio, 1.07; 95% confidence interval, 0.58–1.98). However, interrupted time-series analysis demonstrated a significant decreasing trend in CLABSI incidence following the intervention (rate ratio, 0.89 per month; 95% confidence interval, 0.81–0.97; *p* = 0.01). This trend was observed despite the higher patient severity and increased use of advanced supportive therapies in the post-intervention period. The device utilization ratio and monthly blood culture rate remained unchanged. Avoidance of femoral venous access increased, and adherence to catheter-handling protocols significantly improved. **Conclusions**: A staged, multimodal intervention was associated with a significant decreasing trend in CLABSI incidence over time, suggesting a potential benefit of comprehensive infection prevention strategies in ICU settings.

## 1. Introduction

Central line-associated bloodstream infection (CLABSI) is one of the most common healthcare-associated infections in intensive care units (ICUs), and is associated with substantial morbidity and mortality [1,2]. Reported CLABSI rates in high-income countries range from approximately 0.5 to 2.0 cases per 1000 central line-days [3], whereas substantially higher rates have been reported in low- and middle-income countries, ranging from approximately 1.6 to 44.6 cases per 1000 central line-days [4]. CLABSI has also been associated with attributable mortality ranging from 3% to 25% [5], prolonged hospitalization, and increased healthcare costs [6,7]. The World Health Organization (WHO) has emphasized that up to 70% of healthcare-associated infections are preventable through strict adherence to infection prevention and control measures, highlighting the importance of establishing effective infection prevention and control programs and surveillance systems [8]. In line with this, guidelines recommend a central line insertion and maintenance bundle, including hand hygiene, maximal sterile barrier precautions during catheter insertion, skin antisepsis with 2% chlorhexidine, avoidance of the femoral insertion site when possible, and prompt removal of unnecessary catheters [9,10]. Implementation of such bundled interventions has been reported to reduce CLABSI incidence by up to 66% [11]. However, traditional infection control strategies have primarily focused on technical interventions, which alone may be insufficient to achieve a sustained reduction in CLABSI. Increasing evidence suggests that multifaceted approaches incorporating adaptive or behavioral interventions, such as multidisciplinary rounds, staff education, performance feedback, and continuous monitoring, are essential for improving adherence to infection prevention practices and enhancing patient outcomes [12,13,14,15,16,17].

Therefore, this study aimed to evaluate changes in CLABSI incidence following implementation of a staged multimodal infection control intervention in the ICU using an interrupted time-series (ITS) analysis.

## 2. Results

### 2.1. ICU Characteristics and Baseline Variables

During the study period, ICU admissions were 899 and 1036 in the pre- and post-intervention periods, respectively (Table 1). Altogether, 6379 patient-days and 4333 central line-days were recorded in the pre-intervention period compared to 8428 patient-days and 5951 central line-days in the post-intervention period. Patient severity was higher in the post-intervention period, with higher median Acute Physiology and Chronic Health Evaluation II (APACHE II) scores (16 vs. 17, *p* = 0.02), a higher proportion of patients requiring mechanical ventilation (47.9% vs. 53.8%, *p* = 0.01), and continuous renal replacement therapy (10.3% vs. 15.7%, *p* < 0.001). ICU mortality and length of stay did not differ significantly between the two periods. The distribution of central venous catheter (CVC) types differed significantly between the two periods. The proportion of non-tunneled CVCs decreased (77.5% vs. 68.7%, *p* = 0.003), whereas the use of peripherally inserted central catheters (PICCs) increased (15.8% vs. 25.9%, *p* < 0.001) in the postintervention period. The monthly rate of blood culture sets per 1000 patient-days did not differ significantly between the two periods.

### 2.2. CLABSI Incidence and Temporal Trends

**Figure 1:**
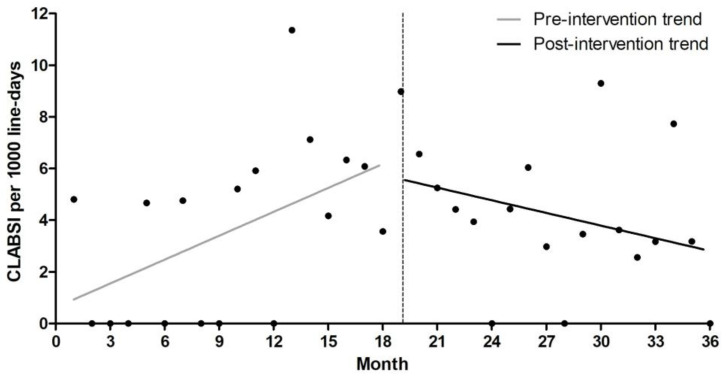
Monthly CLABSI incidence per 1000 central line-days before and after implementation of the multimodal intervention. The dashed vertical line indicates the time point of intervention implementation. CLABSI, central line-associated bloodstream infection.

**Figure 2:**
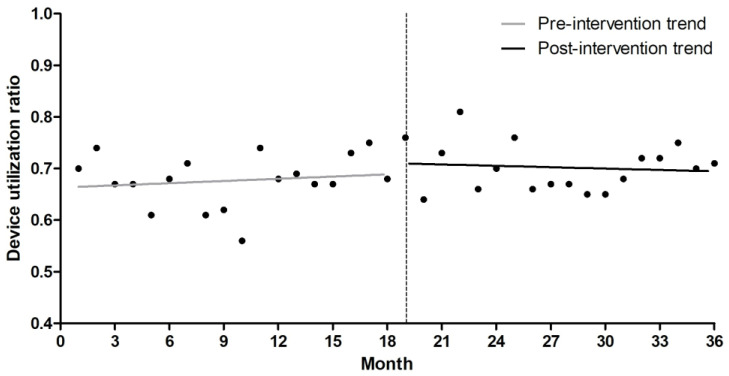
Monthly device utilization ratio before and after implementation of the multimodal intervention. The dashed vertical line indicates the time point of intervention implementation.

Altogether, 17 CLABSI cases were identified during the pre-intervention period, and 25 cases during the post-intervention period (Table 2). The CLABSI incidence was 3.92 and 4.20 per 1000 central line-days in the pre- and post-intervention periods, respectively, with no statistically significant difference between the two periods (incidence rate ratio [IRR], 1.07; 95% confidence interval [CI], 0.58–1.98; *p* = 0.83). Interrupted time-series analysis demonstrated no significant immediate change following the intervention (rate ratio [RR], 0.86; 95% CI, 0.45–1.63; *p* = 0.64). However, a significant decreasing trend in CLABSI incidence was observed during the post-intervention (RR, 0.89 per month; 95% CI, 0.81–0.97; *p* = 0.01), whereas the pre-intervention period showed a significant increasing trend (RR, 1.09; 95% CI, 1.01–1.16; *p* = 0.02) (Table 3, Figure 1). Model diagnostics showed no evidence of significant residual autocorrelation (Durbin–Watson statistic = 2.20; Ljung–Box test *p* > 0.05). No substantial overdispersion was identified (Pearson χ^2^/df = 0.61). In contrast, the device utilization ratio (DUR) remained similar between the two periods without a significant temporal change over the study period (Table 2 and Figure 2).

### 2.3. Changes in Infection Prevention Practices

Compliance with infection prevention practices is shown in Table 4. The proportion of femoral venous access avoidance was higher in the post-intervention period (67.8% vs. 74.2%, *p* = 0.03). Adherence to catheter handling protocols was higher after the intervention (94.6% vs. 98.5%, *p* = 0.01). In contrast, adherence to catheter maintenance protocols was lower in the post-intervention period (96.7% vs. 91.6%, *p* < 0.001). Compliance with hand hygiene, maximal sterile barrier precautions, chlorhexidine skin antisepsis, and aseptic insertion technique remained 100% in both periods.

There were no significant differences in the median duration of CVCs (5 [interquartile range (IQR), 2–13] vs. 7 [IQR, 3–15] days; *p* = 0.05) and the proportion of CVCs maintained for more than 14 days (63/329 [19.1%] vs. 203/872 [23.3%]; *p* = 0.12) between the pre- and post-intervention periods. In the non-tunneled CVCs subgroup, no significant differences were observed in the median catheter duration or proportion of catheters maintained for more than 14 days (Table 4).

### 2.4. Microbiological Characteristics

The distribution of pathogens causing CLABSI was similar between the pre- and post-intervention periods (Table 5). The proportion of patients with candidemia was comparable between the two periods (7/17 [41.2%] vs. 10/25 [40.0%]; *p* = 0.94). There were no significant differences in the proportion of multidrug-resistant organisms (2/17 [11.8%] vs. 4/25 [16.0%], *p* > 0.99) or polymicrobial infections (2/17 [11.8%] vs. 4/25 [16.0%], *p* > 0.99).

## 3. Discussion

This study evaluated the impact of a multimodal infection control intervention on CLABSI incidence in the ICU using a quasi-experimental design and interrupted time-series analysis. Although no significant reduction in CLABSI incidence was observed in the simple pre–post comparison, ITS analysis demonstrated a significant decreasing trend in CLABSI incidence following the intervention. These findings suggest that intervention may have contributed to gradual temporal improvements in catheter-related infection control rather than an immediate reduction in CLABSI incidence.

Previous studies have consistently demonstrated substantial reductions in CLABSI incidence following the implementation of bundled infection prevention strategies [18]. In a large meta-analysis, CLABSI incidence decreased from a median of 6.4 per 1000 catheter-days (IQR, 3.8–10.9) to 2.5 per 1000 catheter-days (1.4–4.8) after insertion and maintenance bundle implementation, corresponding to an IRR of 0.44 (95% CI, 0.39–0.50) [19]. However, the authors emphasized that effective implementation requires multimodal strategies that address behavioral and organizational factors. In the Michigan ICU study by Pronovost et al., a reduction in CLABSI was achieved through evidence-based insertion practices as well as a comprehensive unit-based safety program that incorporated checklists, team leadership, staff empowerment, and feedback [11]. Following this study, many healthcare institutions adopted comprehensive multimodal infection control interventions, including education, performance feedback, checklist-based monitoring, and leadership-driven multidisciplinary rounds, and reported significant reductions in CLABSI incidence [11,12,13,14,15,17,20,21].

However, the magnitude of effect observed in our study was more modest than that reported in several previous CLABSI intervention studies. Unlike earlier studies that demonstrated marked immediate reductions in CLABSI incidence following highly standardized bundle implementation and intensive institutional support, our study did not demonstrate a significant immediate reduction in the simple pre–post comparison. Several factors may explain this discrepancy. First, the post-intervention period included patients with substantially higher illness severity and greater use of supportive therapies, including mechanical ventilation and CRRT, which may have increased the baseline risk of CLABSI. Second, the relatively small number of CLABSI events may have limited statistical power to detect immediate changes. Third, the intervention was implemented in a staged real-world ICU setting rather than as a tightly controlled protocol-driven intervention, which may have resulted in more gradual behavioral and organizational changes over time rather than abrupt reductions in CLABSI incidence. Importantly, the monthly blood culture rate and device utilization ratio remained similar between the two periods, suggesting that the observed temporal decline was unlikely to be explained solely by differences in diagnostic intensity or catheter exposure. In addition, no statistically significant differences in pathogen distribution were identified between the two periods. These findings suggest that the observed reduction in CLABSI incidence was not attributable to suppression of a specific organism or transient changes in the microbial environment, both rather reflected a broader reduction across pathogens commonly encountered in the ICU.

Notably, the distribution of CVC types differed between the two periods, with an increased use of PICCs and decreased use of non-tunneled CVCs in the post-intervention period. PICCs are generally associated with a different risk profile than non-tunneled CVCs, including a lower risk of CLABSI and longer dwell times [22,23]. However, recent meta-analytic evidence indicates no significant infection rates between PICCs and other CVCs in the hospital setting [24]. In addition, PICC use has been associated with fungal bloodstream infections in certain high-risk clinical settings [25]. In our study, *C. albicans* CLABSI was numerically more frequent during the post-intervention period; however, this difference was not statistically significant. Similarly, no significant differences were observed in the proportion of candidemia, multidrug-resistant organisms, or polymicrobial infections between the two periods. Although the relatively small number of microbiological events may have limited statistical power, these findings highlight the need for continued attention to the potential infectious risks associated with prolonged PICC use in ICU patients and warrant further investigation in larger studies.

Consistent with this interpretation, improvements were observed in key process measures. Femoral catheterization is associated with a higher incidence of infectious complications [26]. Accordingly, current guidelines recommend avoiding femoral venous access whenever feasible and preferentially using subclavian or internal jugular access [9,10]. However, in real-world clinical practice, femoral venous access is still frequently used because of procedural convenience, urgent clinical situations, and the need for multiple vascular access sites. In our intervention, the education component specifically emphasized the importance of avoiding femoral catheterization whenever feasible. In addition to catheter site selection, regular reassessment of catheter necessity and reinforcement of catheter maintenance practices were emphasized during weekly infection control team-lead rounds as part of a multimodal intervention strategy. Following the intervention, the proportion of femoral venous access decreased significantly, indicating improved adherence to recommended insertion practices. In addition, adherence to catheter handling protocols improved following the intervention. However, adherence to catheter maintenance protocols decreased. This finding should be interpreted cautiously, as compliance assessments were based on direct observation and checklist-based surveillance, which may have been subject to observer bias and ceiling effects. In addition, the increased frequency and intensity of structured observations and audits during the intervention period may have led to more sensitive detection of previously underrecognized maintenance protocol deviations rather than a true deterioration in practice quality.

This study has several limitations. First, this was a single-center study conducted in a referral hospital, which may limit the generalizability of the findings to other settings. Second, the relatively small number of CLABSI events may have reduced the statistical power of the study and warrants cautious interpretation of the findings. In addition, ICU-specific analyses were not performed because the limited number of CLABSI events was considered insufficient for reliable subgroup comparisons among ICU subtypes. Third, residual confounding factors cannot be excluded. Patient severity and the use of advanced supportive therapies, which would generally be expected to increase the baseline risk of CLABSI, were higher in the post-intervention period. However, the relatively small number of monthly CLABSI events limited the feasibility of incorporating multiple time-varying covariates into the interrupted time-series model. Fourth, although several process indicators were assessed, unmeasured factors related to catheter care and clinical practice may have contributed to the observed outcomes. Compliance assessments were based on direct observation and checklist-based surveillance, which may have been subject to observer bias and potential overestimation of adherence. In addition, detailed fidelity measures for individual intervention components were not systematically collected throughout the study period. Although the preparatory phase before formal intervention implementation primarily involved surveillance and documented optimization, some influence of preparatory activities on clinical practice and outcomes cannot be completely excluded. Finally, as this was a quasi-experimental study, the causal inferences should be interpreted with caution.

In conclusion, a staged, multimodal infection control intervention was associated with a significant decreasing trend in CLABSI incidence over time. This trend was observed despite higher patient severity, increased use of advanced supportive therapies, and greater diagnostic intensity in the post-intervention period, as well as unchanged catheter utilization. These findings suggest the potential role of comprehensive multimodal infection prevention strategies, including improvements in catheter insertion and handling practices, in reducing CLABSI incidence in real-world ICU settings. Further multicenter studies with longer follow-up periods are warranted to confirm these findings and better define the most effective components of multimodal interventions.

## 4. Materials and Methods

### 4.1. Study Design and Population

This study was conducted in the adult ICUs of a 730-bed referral hospital, Chung-Ang University Gwangmyeong Hospital, Gyeonggi-do, South Korea, consisting of 33 medical and surgical ICU beds and 10 beds in the coronary care unit (CCU) and neurological care unit (NCU). All adult patients admitted to the ICUs between 1 January 2023, and 31 December 2025, were eligible for inclusion. Patients who were discharged within 48 h of ICU admission were excluded. An enhanced, multimodal infection control intervention was implemented on 1 July 2024. The study period was divided into pre-intervention (January 2023 to June 2024) and post-intervention (July 2024 to December 2025) periods.

### 4.2. Definitions

CLABSI was defined according to the Korean National Healthcare-associated Infections Surveillance System (KONIS) [27]. Cases occurring ≥48 h after ICU admission or within 48 h before ICU discharge were included. The CLABSI incidence rate was calculated as the number of CLABSI events divided by the total number of central line-days, expressed per 1000 central line-days [27]. The DUR was calculated as the number of central line-days divided by patient-days [27].

### 4.3. Data Collection

Data were retrospectively collected from electronic medical records and infection surveillance databases. Monthly aggregated data were obtained for CLABSI events, central line-days, patient-days, and ICU activity indicators. Compliance with infection prevention practices was assessed using structured audits and observational data routinely collected by the infection control team.

### 4.4. Intervention

**Figure 3:**
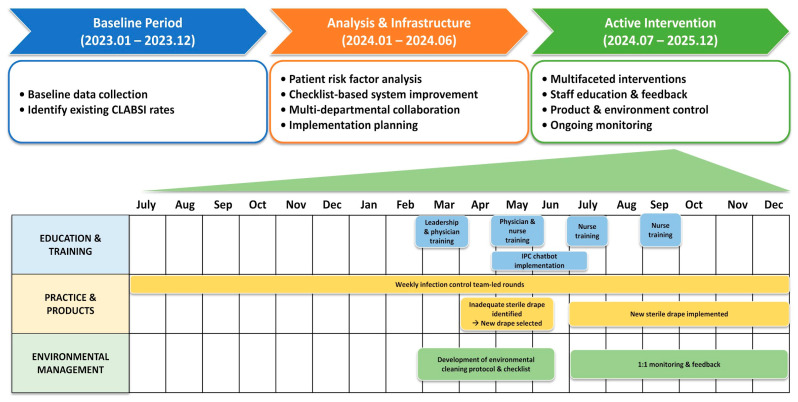
Timeline and components of the multimodal infection prevention and control intervention.

The intervention consisted of a comprehensive multimodal infection control program implemented in July 2024 (Table 6 and Figure 3). From January 2023 to December 2023, routine surveillance data were collected to establish the pre-intervention CLABSI incidence and characterize baseline catheter-related practices. An in-depth analysis of CLABSI risk factors was conducted from January 2024 to June 2024. In addition, the electronic documentation system for catheter insertion and maintenance was refined, including improvements to checklist-based data entry, to enhance data completeness and timeliness. This preparatory phase primarily involved surveillance enhancement and optimization of documentation systems rather than active implementation of infection prevention measures. CLABSI surveillance methods and diagnostic definitions remained consistent throughout the study period, and no major ICU-wide infection control policy changes or concurrent CLABSI-targeted interventions outside the described multimodal intervention framework were introduced during the study period. The intensive intervention phase (July 2024 to December 2025) consisted of a comprehensive multimodal program with three key components: (1) education and organizational engagement, (2) optimization of catheter insertion and maintenance practices, and (3) reinforcement of environmental hygiene. Educational interventions included repeated structured training and re-education for physicians, nurses, and leadership staff regarding CLABSI prevention practices, catheter bundle adherence, catheter handling, and environmental infection control. Weekly infection control-led rounds were conducted to assess catheter necessity, dressing condition, and prolonged catheter use using standardized checklist-based surveillance protocols. An infection control chatbot was also introduced to provide real-time access to guideline-based recommendations. To optimize catheter-related practices, adherence to maximal sterile barrier precautions during catheter insertion was reinforced, and insertion kits and sterile supplies were standardized. Weekly infection control team-led rounds were conducted to assess catheter necessity, dressing conditions, and prolonged catheter use, with recommendations for removal or replacement when appropriate. Checklist-based maintenance surveillance also included assessment of dressing integrity and cleanliness, stopcock closure, contamination of catheter connection sites, insertion-site abnormalities, and adherence to recommended exchange intervals for infusion sets and three-way stopcocks. Environmental hygiene was strengthened through the implementation of standardized ICU cleaning protocols, along with regular monitoring and feedback to improve compliance.

### 4.5. Outcomes

The primary outcome was the CLABSI incidence rate per 1000 central line-days. Secondary outcomes included DUR, ICU activity indicators, and compliance with central line insertion, maintenance, and handling practices. In addition, temporal trends in CLABSI incidence were evaluated using an ITS analysis.

### 4.6. Statistical Analysis

Continuous variables were expressed as median and IQR, and categorical variables were expressed as counts and percentages. The CLABSI IRR between the pre- and post-intervention periods was calculated using aggregated CLABSI events and total central line-days, with 95% CIs. Monthly DUR and monthly blood culture set rates were compared using the Mann–Whitney U test. Categorical variables were compared using the chi-squared test or Fisher’s exact test, as appropriate. ITS analysis was performed using a segmented Poisson regression with monthly CLABSI counts as the outcome and the logarithm of central line-days as an offset variable. ITS analysis was selected because it enables evaluation of both immediate and gradual temporal changes following implementation of a system-level intervention in a real-world setting where randomized allocation was not feasible. The model included terms for time (pre-intervention trend), intervention (immediate level change), and post-intervention time (trend change after intervention). Residual autocorrelation was assessed using Durbin–Watson statistics and Ljung–Box tests, and robust standard errors were applied in the segmented Poisson regression model. Overdispersion was evaluated using the Pearson chi-square statistic divided by the residual degrees of freedom. Because no clear seasonal pattern was identified on visual inspection of monthly incidence data, seasonality terms were not incorporated into the final model. The results were reported as RRs with 95% CIs. A two-sided *p*-value < 0.05 was considered statistically significant. Statistical analyses were performed using IBM SPSS Statistics version 23.0 (IBM Corp., Armonk, NY, USA) and R software version 4.4.1 (R Foundation For Statistical Computing, Vienna, Austria). Figures were generated using GraphPad Prism version 5 (GraphPad Software, San Diego, CA, USA).

## Figures and Tables

**Table 1:** ICU activity and clinical characteristics during the study period.

Variable	Pre-Intervention	Post-Intervention	*p*-Value
Age, median (IQR)	71.0 (59.0–80.0)	70.0 (59.3–80.0)	0.80
Male, *n* (%)	504 (56.1)	591 (57.0)	0.66
ICU admissions	899	1036	
Patient-days	6379	8428	
Central line-days	4333	5951	
ICU mortality, %	13.8	15.7	0.23
ICU length of stay, median (IQR), days	6 (4–12)	6 (4–14)	0.14
APACHE II score, median (IQR)	16 (8–22)	17 (10–23)	0.02
Mechanical ventilation, %	47.9	53.8	0.01
CRRT, %	10.3	15.7	<0.001
ECMO, %	3.4	4.0	0.56
Central venous catheter distribution, *n* (%)			
Non-tunneled	255 (77.5)	599 (68.7)	0.003
Tunneled	12 (3.6)	23 (2.6)	0.35
Peripherally inserted central catheter	52 (15.8)	226 (25.9)	<0.001
Implanted port	10 (3.0)	24 (2.8)	0.79
Monthly rate of blood culture sets per 1000 patient-days, median (IQR)	321.3 (298.1–384.2)	335.2 (303.0–364.7)	0.84

IQR, interquartile range; ICU, intensive care unit; APACHE II, Acute Physiology and Chronic Health Evaluation II; CRRT, continuous renal replacement therapy; ECMO, extracorporeal membrane oxygenation.

**Table 2:** CLABSI incidence and device utilization ratio.

Outcome	Pre-Intervention	Post-Intervention
CLABSI cases	17	25
CLABSI incidence (per 1000 line-days)	3.92	4.20
DUR, median (IQR)	0.68 (0.67–0.71)	0.70 (0.67–0.73)

CLABSI, central line-associated bloodstream infection; DUR, device utilization ratio; IQR, interquartile range.

**Table 3:** Interrupted time-series analysis of CLABSI incidence.

Variable	RR	95% CI	*p*-Value
Pre-intervention trend (per month)	1.09	1.01–1.16	0.02
Immediate level change after intervention	0.86	0.45–1.63	0.64
Post-intervention trend (per month)	0.89	0.81–0.97	0.01

Rate ratios were estimated using a segmented Poisson regression model with an offset for central line-days. The model included terms for time, intervention, and post-intervention time. Robust standard errors were applied. Residual autocorrelation and overdispersion were assessed using Durbin–Watson statistics, Ljung–Box tests, and the Pearson chi-square statistic divided by residual degrees of freedom. CLABSI, central line-associated bloodstream infection; RR, rate ratio; CI, confidence interval.

**Table 4:** Compliance with central line insertion, maintenance, and handling practices.

Indicator	Pre-Intervention (%)	Post-Intervention (%)	*p*-Value
Median duration of non-tunneled central venous catheter	5 (2–9)	5 (2–10)	0.33
Prolonged non-tunneled CVC use (>14 days)	28/255 (11.1)	71/599 (11.9)	0.72
Catheter insertion protocol			
Avoidance of femoral venous access	223/329 (67.8)	647/872 (74.2)	0.03
Hand hygiene compliance	324/324 (100%)	557/557 (100%)	N/A
Maximal sterile barrier adherence	324/324 (100%)	557/557 (100%)	N/A
2% Chlorhexidine skin antisepsis	324/324 (100%)	557/557 (100%)	N/A
Aseptic insertion technique	324/324 (100%)	557/557 (100%)	N/A
Catheter maintenance protocol adherence	780/807 (96.7%)	1000/1092 (91.6%)	<0.001
Catheter handling protocol adherence	330/349 (94.6%)	337/342 (98.5%)	0.01

CVC, central venous catheter.

**Table 5:** Distribution of pathogens causing CLABSI in the pre- and post-intervention periods.

	Pre-Intervention (*n* = 17)	Post-Intervention (*n* = 25)
Gram positive cocci		
*Staphylococcus aureus*	0	1
*Enterococcus faecalis*	2	2
*Enterococcus faecium*	1	3
Gram-positive rods		
*Corynebacterium striatum*	1	0
*Clostridium innocuum*	1	0
Gram-negative rods		
*Escherichia coli*	1	1
*Klebsiella pneumoniae*	1	3
*Klebsiella oxytoca*	0	1
*Klebsiella aerogenes*	1	0
*Enterobacter cloacae*	1	1
*Citrobacter freundii*	0	1
*Morganella morganii*	0	1
*Pseudomonas aeruginosa*	1	1
*Stenotrophomonas maltophilia*	1	0
*Burkholderia cepacia*	0	1
*Aeromonas caviae*	0	1
*Cupriavidus* spp.	0	1
Fungi		
*Candida albicans*	2	6
*Candida glabrata*	3	2
*Candida tropicalis*	3	3
*Pichia kudriavzevii*	0	1

Polymicrobial infections were identified in 2 cases during the pre-intervention period and in 4 cases during the post-intervention period. CLABSI, central line-associated bloodstream infection.

**Table 6:** **Components of the multimodal infection control intervention**.

Component	Description
Education and organizational engagement	Leadership-focused infection control education; structured training for physicians and nursing staff; implementation of an infection control chatbot for guideline reinforcement
Catheter insertion and maintenance optimization	Strengthened bundle adherence monitoring; product optimization to enhance maximal sterile barrier compliance; weekly infection control team rounds for catheter necessity and maintenance assessment
Environmental hygiene reinforcement	Development and implementation of standardized ICU environmental cleaning protocols; monitoring, feedback, and performance evaluation of disinfection practices

ICU, intensive care unit.

## Data Availability

The datasets generated and analyzed during the current study are not publicly available due to patient confidentiality but are available from the corresponding author on reasonable request.

## Abbreviations

The following abbreviations are used in this manuscript:

CLABSI	Central line-associated bloodstream.
ICU	Intensive care unit.
WHO	World Health Organization.
APACHE II	Acute Physiology and Chronic Health Evaluation II.
CVC	Central venous catheter.
PICC	Peripherally inserted central catheter.
IRR	Incidence rate ratio.
CI	Confidence interval.
ITS	Interrupted time-series.
DUR	Device utilization ratio.
IQR	Interquartile range.
